# Implementing turnaround strategies as an entrepreneurial process

**DOI:** 10.1007/s11365-022-00810-9

**Published:** 2022-10-01

**Authors:** Peter Baliouskas, Juan Llopis, Jose Gasco, Reyes Gonzalez

**Affiliations:** Department of Business Organization, University of Alicante, Alicante, Spain

**Keywords:** Turnaround strategy, Entrepreneurial process, Financial crisis, Greece Economy

## Abstract

Recovery strategy belongs to the group of rescue/reversing business strategies and is used mainly by companies facing financial or other problems which they are trying to overcome. Moreover, this strategy is alternatively used by companies wishing to prevent future problems. The overall objective of the recovery strategy is to return of this sluggish situation in terms of acceptable levels of profitability. The implementation of turnaround strategy achieved through the implementation of proper planning and specific procedures (processes) like: Change management, divestitures of specific assets (divestment), cost reduction (cost reduction—operating and others), and strategic acquisitions. This strategy is so drastic, that in many senses it is like creating a new company, so it has many similarities to an entrepreneurial process. Obviously, there is a previous experience by managers, but when implementing this radical strategy, they must think in developing something new. The main objective of this paper is to explain how to implement a successful turnaround strategy during a recession phase of the economy. To analyze the turnover strategy, we developed a survey to 152 trading and industrial Greek companies that represent more than 3% of the Greek GDP. The study examines the role of successful turnaround based to whom formulated the strategic plan of the company, investigating the knowledge of the Greek managers on the tactics implemented during a turnaround plan. The paper offers information for management practitioners to understand how to implement a turnaround strategy in a turbulent economic environment, and which tactics apply.

## Introduction

In this study, we previously analyze the literature in relationship with turnover strategy and management, trying to overview the nature, applicability, stages, and consequences. After that, a description of the financial crisis in the Greece economy is necessary to understand the need and pertinence of this research.

With the help of descriptive statistics, we analyze the demographic characteristics of those who participated in the survey and the companies they represented. We record the results that their business factors were influenced such as their strategic plan, their turnover, their ROI and their ROA. We recorded the external and internal factors that influenced them, the results that were achieved and the tactics that were implemented.

In this sense, with the help of statistical models, we analyze the correlation of many factors for the index of ease/difficulty of application of tactics that were applied as well as for the correlation of the factors that brought successful results. We also study the importance which has for successful results by whom the strategic plan is formulated and implemented. In addition, conclusions are described for the right order of turnaround tactics implemented based on the experience and knowledge of the managers of Greek companies. Pearson Chi square tests, spearman correlation coefficient models, Kruskal Wallis test (analysis of variance) and Binary logistic regression were used to analyze all of them. Additionally, we use factor analysis, cluster analysis implemented and analyzed with spearmen coefficient correlation test and ANOVA analysis.

The study's major goal is to establish a theoretical framework that explains how turnaround strategy implemented during a recession phase of the economy and how economic crisis affected on strategy plan of the companies, turnover, results, ROI & ROA.

In a more specific way, the objectives consist in analysing the impact of economic crisis on Greek companies taking into consideration the strategic plan, turnover and economic results; determine what extent specific tactics were applied and the results; describe the results of Greek companies after the implementation of the measure; and determine the perception of Greek managers according to the tactics that they had to apply to overcome a crisis period.

To answer these questions, we divide the paper in the next sections: study of the concept of turnaround strategy and entrepreneurial process, the analysis of the financial crisis of 2009 in Greece, the methodology of the research, results (with descriptive statistics and statistical analysis), and conclusions (including limitations and future lines of research).

## Turnaround strategy and entrepreneurial process

Generally, turnaround strategy is a corporate strategy designed to save a company which is suffering loss. A turnaround strategy is a series of moves made by an organization's management that helps return the organization to profitability. Turnaround is not something new in the business world. In fact, Gotteiner et al. (2019) observe that corporate turnaround research has developed dramatically over the last past three decades. Probably, the first use of the term turnaround is made by Schendel et al. (1976) and is defined as “a significant recovery of business performance after a significant decline” or “a transition from decline to an upward trend”. Organizational turnaround processes are primarily concerned with business renewal. Turnaround management activities thus revolve around a review of management practices, activity-based costing, and SWOT analysis to determine the root causes of a company's failure (Thompson et al., 2014). Thain and Goldthorpe (1989) use the definition “*Reversing Performance from Decline and Failure to Recovery and success”* to define the meaning of the term turnaround (Charalabidis, 2011). Turnaround strategy means to turn a losing company into a profitable one. This is to make the company more profitable again. In this sence, Cater III and Schwab (2008), Schmitt and Raisch (2013), Tangpong et al. (2015), Schweizer and Nienhaus (2017), and Bhattacharyya and Malik (2020) affirm that the main purpose of implementing a turnaround is to change a negative company to a positive one.

Without a suitable turnaround strategy to support it, a sick company will shut down in due course. It is a remedy for industrial instability. Turnaround is a restructuring procedure. After this, a decline company can be transformed into a profit-making one, by implementing a standardized method. It attempts to improve weaknesses to help companies that are sick, weak, and not profitable to recover. Ghazzawi (2018) sustains that turnaround is focused on managerial cognition, efficiency, and relevant performance criteria and based on identifying causes of decline by top management. In addition, Nyagiloh and Kilika (2020) concludes that a turnaround strategies play critical roles that are concentrated in looking at the processes geared towards corporate renewals through analysis and planning mechanisms to return troubled firms to solvency.

This is a complete U-turn of the relevant planned strategic economic transition. Gopal (1991) provides a more detailed interpretation of the term: “the process by which companies reduce their losses and achieve increased profitability, the process of converting a *failed* organization into a successful one, a *poor* one into a satisfactory one, a *patient one* to a profitable company”. In this case, the focus is on profitability. Rico et al. (2021) affirm that a key assertion in the turnaround literature is that when survival is threatened, it is necessary to undertake asset and cost retrenchment strategies that stabilize the performance decline and provide a base for survival and recovery. Similarly, Bruton and Wan (1994) interpretation focuses on financial performance: “recovery is the reversal of the decline of an organization's financial performance”. Barker and Barr (2002) generally define the recovery effort as “an orchestrated and organized effort by the company's top management to respond to corporate performance problems”. Barker and Duhaime (1997), Gatti (2002), Chowdhury (2002), Sheppard and Chowdhury (2005), Charalabidis (2011), and Al-Turki et al. (2019) state that a successful recovery takes place when a company is going through a performance decline that threatens its existence for a period of years but capable of reversing the decline in performance, ending the threat of survival, and achieving sustainable performance.

The American Turnaround Management Association states that a recovery plan is necessary after “a period of reduced profits, high costs and/or failure to meet financial obligations” (Oliver & Fredenberger, 1997). The threat of survival is also introduced by Pandit (2000). Balgobin and Pandit (2001) understand turnover as “the recovery of corporate financial performance after a decline threatening the existence of the company”. Similarly, Sheppard and Chowdhury (2005) report that “recovery is defined as the action taken to prevent the occurrence of financial disaster”.

The causes of corporate decline are one of the most explored elements of a turnaround strategy (Pandit, 2000). More specifically, O’Neill (1986b), Pearce and Robbins (1993) and Butar et al. (2019) refereed that causes of decline affect the solutions which finally adopted, and Scherrer (2003) argues that understanding the causes of decline is the first step to recovery. This view is supported by the existence of evidence for the ability of companies to be rescued if they identify, handle and reverse the causes of decline (Harker, 1996, and Boyne & Meier, 2009), and by the fact that the analysis of the causes of decline is the starting point of various recovery models as defend O'Neill (1986a), Gopinath (1991), Chowdhury (2002) and Lohrke et al. (2004).

Large-scale empirical research, such as that of Robbins and Pearce (1992), shows that the choice of recovery strategy is influenced by the cause of decline or, more correctly, management's perception of the cause of decline.

So, the reasons that a company could be led to decline are distinguished into internal and external. When a company comes to decline due to internal reasons it is probably easier to apply appropriate tactics and achieve a successful turnaround. This is because the whole arrangement of the situation depends on the management team which will have to correct an internal problem such as poor production process, lack of financial control, marketing issues, etc. External reasons for decline are more difficult to be managed and the time need to be balanced and succeed on a turnaround strategy is much longer. The reason for that is because decline related to external factors such as falling demand, increased competition, reduced GDP, government restrictions, are not controlled by the company’s managers. Based on the above context Slatter and Lovett (1999) argues that it is much easier to achieve turnaround in companies which experience a state of decline due to internal causes in comparison to those companies which the decline cause is by external factors.

Balgobin and Pandit (2001) in the “stages in turnaround process” referred that according to existing literature, there are 5 stages identified in the turnaround process. More specifically they are distinguished decline and crisis; triggers for change; recovery strategy formulation; retrenchment and stabilization; and, return to growth. However, the dominant categorization based on the literature is: a) management change, b) evaluation stage, c) emergency stage, d) stabilization stage, e) return to growth stage. The number of possible strategies is extensive and diverse. It is also expected that each strategy will have a different degree of suitability, receptivity and applicability, depending on the context faced by each company and, according to Gopal (1991) and Schoenberg et al. (2013), there are no standard strategies for company recovery. O'Neill (1986a) proposes, based on a review of successful and failed corporate recovery efforts in the US, the classification of strategies into 4 groups: a) administrative, b) retrenchment, c) development and d) restructuring. The management strategies include the change or the reorganization of the management team and the strengthening of the morale of the employees.

In most turnaround management studies; poor management is one of the most important factors in decline and therefore in most turnaround cases a change of leadership is necessary. Grinyer, McKiernan and Yasai-Ardekani (1988) found that most successful turnaround procedures implemented by companies were associated with a change of management team as opposed to failing to achieve recovery which had not led to a change of management. According to O'Neill (1986a), shareholders expect the business leadership to take control of the organization and keep top managers responsible for the performance of the company. During the decline, the leader's position was critical (Abebe, 2010; Abebe et al., 2011; O’Kane & Cunningham, 2012). It is therefore common practice to change management. Moreover, according to Chan (1993); Bibeault (1982); Castrogiovanni et al. (1992); Chen and Hambrick (2012); and Cassells (1992), the change of CEO is a very important step for a successful turnaround strategy. There are several reasons for such replacements: current management holds a number of strong and hurtful corporate beliefs that have caused loss of vision, passivity or failure (Arogyaswamy et al., 1995; Daily & Dalton, 1995; Gopinath, 1991; Nystrom & Starbuck, 1984; Schuler & Jackson, 1987).

On the other hand, some researchers believe that a successful turnaround can also be accomplished without such management adjustments (Clapham et al., 2005; Schreuder et al., 1991). Anyway, since the leadership was responsible for the loss, it is very unlikely that he or she would be able to turnaround the organization. Furthermore, a change in leadership would send a clear message to stakeholders (particularly banks and creditors) that something is being done to turnaround the company, resulting in their continued support (Slatter & Lovett, 1999; Sudarsanam & Lai, 2001).

According to Bibeault (1982), the turnaround process is divided into two stages: primary and secondary. The primary targets of driving declined businesses of sustainability and positive cash flow. Retrenchment activities such as divestment, product removal, and headcount reductions are effective methods for achieving these goals. The advanced stage of turnaround is focused on development and growth. Acquisition, new product, new business, and increased market penetration are effective strategies for achieving these goals.

At this point, we must say with Probst and Raisch (2005), Filatotchev and Toms (2006), and Furrer et al. (2007) that no ideal model of turnaround management exists due to the variations in turnaround situations (depending on the causes, stages, and magnitude of crises). Anyway, Pandit (2000) sustain that the option of turnaround strategy is crucial. This author argue that the choice of turnaround strategy and behavior is influenced not only by the particular causes of the crisis and their magnitude, but also by stakeholder behaviors, environmental factors (such as market characteristics or macroeconomic conditions), and previous corporate strategies.

The first turnaround model was formulated by Bibeault (1982). This model is divided into five basic stages. The first stage concerns the change of executives (change management). The second stage is evaluation and identification of the problems. The third stage is that of emergency. At this phase the goal is to stop the negative flows. A list of alternative actions is drawn up, such as lending, for example, against the sale of a fixed asset, disinvestment of loss-making parts, and cuts in unnecessary expenses. The fourth stage is that of stabilization. During this phase, the main target is the increase of profitability. The company now focuses on the core business and planning investments to improve efficiency. The fifth and final phase is the return to growth. At this phase the company aims to change internally the structure, but also to focus on more profitable markets and products.

Another option is the model of Balgobin and Pandit (2001), in summary, the 5 stages of this model are as follows: a): Decline and crisis b): Triggers of change c) Formulation of recovery strategies d) Restriction and stabilization e) Return to growth. In 2002, Chowdhury developed a new model based on four criteria-assumptions: a) the motivation to implement recovery actions arises after a prolonged decline in performance, b) recovery is a series of activities related to the internal and the external context, c) the activities are undertaken and executed decisively and d) the combination of the first three criteria typically extends over a period of time. The 4 stages of the model are the following a) Decline b) Initiation of response c) Transition d) Outcome. Other models are the proposed by Scherrer (2003), Charalabidis (2011), Robbins and Pearce (1992), Pearce and Robbins (1993), Smith and Graves (2005), and Lohrke et al. (2004). According to our perspective, the main distinction that determines the recovery model for a company which is in a turnaround situation is the investigation of the causes that led the company to this situation, as well as the severity of the situation itself. We believe that different causes of decline require different treatments and customized models. There is no model that is a panacea and can be applied on every case.

We can also add that in many previous studies, authors supported that the retrenchment phase is very important for a successful turnaround: Bibeault (1982), Heany (1985), Altman (1983), Ansoff (1977), Goodman (1982) Slatter (1984), Bruton et al. (2003), Makheti and Nyakweba (2016) and Panicker and Manimala (2015) and Amankwah-Amoah et al. (2018).

Based on the results after the implementation of a turnaround strategy, Slatter and Lovett (1999) group companies into 4 categories. 1) Non-recoverable: These businesses would not be able to thrive in the near term. 2) Short-term survival: Companies in this group may have succeeded in adopting restructuring process plans that typically seek to reduce expenses and raise sales in the short term. 3) Sustained survival: This group includes companies which have successfully reversed their fortunes; nevertheless, external conditions such as market decline and scarce capital hinder their ability to expand further. 4) Sustained recovery: This group includes businesses that have made a real and prosperous recovery.

Turnaround strategy is a critical decision and authors as Collet et al. (2014), Kraus et al. (2013), Pan and Chen (2014), Panicker and Manimala (2015), Abebe and Tangpong (2018), Ghazzawi (2018), and Richa and Ashok (2022) consider its similarities with the entrepreneurial process. In this sense, Osiyevskyy et al. (2021) argue that the idea is to find successful pathways through crisis by priming the managerial decision-making towards either entrepreneurial thinking. Obviously, there is a previous experience by managers, but when implementing this radical strategy, they must think in developing something new. In a particular way, Shahri and Sarvestani (2020) consider a business model innovation functions as a critical practice of turnaround strategy in the decline period. More direct is Omorede (2021) arguing that while experiencing the cost of firm failure, entrepreneurs seek for means to recover. In doing so, they seek for strategies to cope with their failures and make sense of the situations that led such failure. We will focus on this paper in the particularities of implementing turnaround strategies.

## The financial crisis of 2009 in Greece

This crisis caused a major recession, was the worst economic collapse to hit the world since the Great Depression of 1929, and it created chaos on financial markets all around the world. The crisis, which was triggered by the burst of the US real estate bubble, culminated in the failure of Lehman Brothers (one of the world's largest investment banks), the near collapse of several financial firms and corporations, and the need for extraordinary levels of government assistance. It took a decade to get back to normal, during which time millions of jobs were lost as well as billions of dollars in revenue.

Following World War II and the civil war that concluded in 1949, Greece's economic efficiency was among the most spectacular in the world, not only in Europe, but also throughout the rest of the world. This continued until the beginning of the 1970s when the first oil crisis hit in the 1970s, Greece suffered a setback but rebounded rapidly. After seven years of dictatorship, the Republic was restored in 1974 and in 1981 Greece joined the EEC. The country, however, experienced a period of stagnating inflation and significant growth of public debt following the second oil crisis, which persisted all over 1980s. In 1990, Greece embarked on a program of fiscal consolidation and structural changes to position the country for potential membership in the European Union's single currency system. Greece was one of the countries that signed the Maastricht Treaty in 1991, and it was only a year later that it was admitted to the Eurozone as a member state. Over the course of the 1990s, economic growth steadily rebounded, inflation gradually decreased, and the public debt as a percentage of GDP reached a stable level. The era from 2001 and 2008, when Greece first joined the Eurozone, was often seen as a “golden age” for the economic growth of the country. Rate of growth continued to rise, inflation remained moderate, although somewhat higher than the euro area average, and unemployment continued to decline. When measured in percentage of GDP, public debt remained at around 100 percent, which was greater than the euro area average, although there was no threat of an insolvency crisis.

In mid-2010 and after the revelations that Greece's budget deficit closed for 2009 at levels above those that would make public debt sustainable, the Greek government was also unable to raise funds from the markets at fair interest rates for financing the fiscal deficit and refinancing the debt. This analysis can be expanded in Alogoskoufis and Featherstone (2021), Afonso et al. (2015), and Hyz (2019). This resulted in the immediate risk of bankruptcy and default of the Greek State.

Maris et al. (2021) states that we cannot understand the severity and duration of the economic crisis in Greece if we dot analyze the crisis in terms of the political perspectives that ultimately highlight not only the restrictions imposed, but the general culture which emerges within the country regarding the operation and effectiveness of its political and economic institutions. Most worrying was the gradual increase in the primary deficit, which in 2009 reached 10.2% of GDP, although this is partly due to the reduction of tax revenues from the current crisis. It is worth mentioning the fact that the large budget deficits characterize almost all economies in the period 2008–2010, although not to the extent that they inflated in Greece. The country was now in a typical situation of “twin deficits”, i.e., the simultaneous existence of a deficit in both the budget balance and the current account balance, while public debt was equal to 127% of GDP and private debt was constantly growing.

The government strived to reestablish the country's validity in international markets to gain interest rate reductions resulted in initiatives to minimize public spending that ended in failure to restore the negative climate. Consequently, Greece sought assistance from the International Monetary Fund, the European Union, and the European Central Bank, which collaborated to establish a cooperative assistance framework for the country. The announcement of the recourse to the support mechanism was made on April 23, 2010, by the Prime Minister George Papandreou. The funding from the support mechanism was made under the conditions that Greece will take fiscal consolidation measures. This financing avoided the immediate risk of bankruptcy of Greece, which would have uncontrollable consequences for the entire euro system. The problems of the Greek economy were the high debt, the borrowing rates, the high unemployment, and the low competitiveness, which forced the state to increase the expenses for the maintenance of the economy. These factors are examined by Ozturk and Sozdemir (2015), Pazarkis et al. (2018), and Mamatzakis et al. (2022).

The crisis was very impacting not only in the economy of Greece, but also in the society. For example, increasing the number of depressions and suicides (Economou et al., 2016), increasing problems with gambling (Economou et al., 2019), less services in the public health care system (Ifanti et al., 2013; Kotsiou et al., 2018), and less energy consumption by the population (Santamouris et al., 2013) to mention some effects. At the same time, a large number of solidarity cases happened to respond to the crisis (Clarke et al., 2016).

Lack of investment and high market prices were also a problem. Unemployment has been on the rise since September 2008 and GDP has been declining, with a deficit of € 717 m (-0.3%) within four months, leading to discussions about a debt refinancing problem. Measures to boost the economy were announced in mid-August. The real annual budget deficit of 15.6% for 2009 meant that within a year the public debt increased from around 110% to over 125% as a percentage of GDP. This is because due to the international financial crisis, but also to other factors, there has been stagnation of GDP and recession since 2008. Therefore, the annual deficit was simply added to the debt. The denominator (GDP) was now stagnant. Due to these unprecedented conditions for the Greek economy, with a GDP of 237 billion in 2009 to 179 billion in 2014 and unemployment from 9% in 2009 to 27% in 2015 (Hellenic Statistical Authority) many companies faced a decline situation and straggled to survive.

## Methodology of the research

In this research, the main goal is to find out what happened to companies in the commercial and industrial sector in Greece during the years 2010–2016 and the extent of the effects of the financial crisis on key factors such as their strategic plan, their turnover and their results and ROI. In addition, to understand the reasons why managers believe that their companies have been affected by the measures they have taken, the tactics they have implemented and whether these tactics have brought successful results. Important for our research was to find out if the differences in tactics mentioned in the literature are easier to apply during a recession phase of the economy.

Our sample consists of 152 participants in our research. Approximately 450 questionnaires were distributed, and 152 companies answered back, most of them by personal contact—interview. Companies in Greece (there are few exceptions) are registered in the GEMI (general commercial register) which is under the authority of the Ministry of Development and Investment. GEMI is the Hellenic Register of Commercial Publicity, as defined in European directives on company law (Directive 2009/101 / EC) and the Electronic Database for Entrepreneurship in Greece, from which information for any company could be obtained from the Public Administration, third parties, banks, and other legal entities.

In Greece companies are distinguished a) according to their size, (law 4308/2014), b) depending on the industry in which they operate and c) based on their legal form. Based on their size divided into the following categories: a) Large companies: Those with more than 40 million turnovers and more than 250 employees, b) Medium-sized enterprises: Those with a turnover of 8–40 million and employing 50–250 employees, c) Small businesses: Those with a turnover of less than 8 million and employing less than 50 people. Depending on the industry in which they operate, companies are classified into a) Commercial, b) Industrial, c) Tourist, d) Banking, e) Services, f) Construction, g) Shipping, etc. Finally based on their legal form companies in Greece can be a) General Partnership, b) Limited Partnership, c) Limited Liability Company, d) Public Limited Company (PLC). Also, there is a new form of company which is called Private Capital Company (PC) made by the law 4072/2012. The establishment of these companies started after the outbreak of the crisis and that is why we did not include such companies in the sample.

The criteria used to share the questionnaires were a) the company to be operating in Greece b) to belong in the commercial (trading) or industrial sector. Additionally, we tried to cover companies of all sizes, from all legal forms and from basic geographical areas. We had also in my mind to collect questionnaires from different business sectors such as electricity, water supply, detergents, metallurgy, dairy industry etc. Moreover, we wanted to collect questionnaires from companies which are listed in the stock exchange and the publication of their data is mandatory strictly, every six months.

We tested ten surveys to be sure that managers could understand the questions without any problem. The survey was submitted-conducted from September 2018 to November 2020. Finally, we used a sample of 152 Greek companies. An online questionnaire was sent by e-mail both to companies listed in ASE and to companies which were not listed in the stock exchange to cover both categories as mentioned before. In many cases we realized an interview to fill the questionnaire immediately because the response rate was very low due to limited time of the managers. Some of the respondents did not answer all the questions and that is why in some cases the sum of the answers is not 152.

In addition, there was great difficulty because our research questionnaire touches on sensitive issues concerning business and confidentiality. Anonymity both company and the respondent, was a key requirement for obtaining the answers.

We tried to cover all the categories regarding the small, medium, and large size. So, our sample is allocated to 35% small companies, 24% medium size companies and 41% large and very large companies; 49% belongs in the industrial sector and 51% in the trading sector. Additionally, we tried to cover geographically the entire Greek territory. In Greece most companies in terms of their headquarters are concentrated in the 2 major cities Athens and Thessaloniki. Nevertheless, we sent questionnaires and tried to reach companies from all over the territory. Finally, the questionnaires collected represent companies that are located 56% in Central Greece (where Athens also belongs), 39% in Northern Greece (where Thessaloniki also belongs) and 5% in southern Greece. So, the data we gathered are from small, medium, and large companies in the commercial and industrial sector and we believe that our sample can give us reliable results if we consider that the total turnover of companies that responded is close to 3% of the country's total GDP. We chose to deal with industrial and trading sector since these sectors affected dramatically by the crisis. Based both on the research data of the Foundation for Economic & Industrial and Stat Bank, the industrial sector in Greece has suffered a spike in losses as recorded in the articles of electronic version of newspapers (Kathimerini 12/7/2011, Newsbob 1/10/2012) (Newsroom, 2011, 2012). The reason, especially in manufacturing companies, was the significant increase in costs by 9.4%, leading to a decrease in gross profits by 8.4%. In addition, the industrial companies also showed increased operating expenses (exceeding the respective gross profits in 2011), resulting in the greater deterioration of the operating result (already loss-making since 2010). Eventually, the sector's losses more than doubled. Trading sector, resulting in a 4.5% decrease in their sales in 2011, with a sharp decline in gross profits of 6.9%, due to the squeezing of profit margins as compensation for the limited demand.

Qualitative variables are expressed as absolute (N) and relative frequency (%) in each category of the variable. A Pearson chi-squared test was performed to assess possible differences that may exist in the questionnaire questions in relation to some demographics’ characteristics. The underlying statistical assumptions in this case are:

Ho: there is no relationship between the two variables.

H1: There is relationship between the two variables.

If p-value is less than 0.05 (p < 0.05) the zero hypothesis is rejected, resulting in a statistically significant relationship between the two variables.

The Spearman correlation coefficient was used to assess the linear correlation between two quantitative variables. This measure can take a range of values from + 1 to -1. A value of 0 indicates that there is no association between the two variables. Values close to + 1 indicates a positive association and values close to -1 indicates a negative association.

Additionally, Kruskal Wallis test was implemented to investigate statistically significant differences between some groups of specific variables and also Binary logistic regression applied.

A factor analysis was conducted in order to reduce the number of initial variables into fewer factors. The variables used were the questions “Please, indicate the extent to which you believe that the following external factors were responsible for the crisis situation that your company faced” and “Please, indicate the extent to which you believe that the following internal factors were responsible for the crisis situation that your company faced” with possible answers (1) Entirely Disagree to (7) Extremely agree.

We used these factors in a Spearman correlation coefficients test for the examination of the following question “Please, indicate the extent to which you believe that country economic situation affected negatively to the following operations-indicators” (Strategic plan, Turnover, Results of your company, ROI, ROA) which are very important to define a crisis or a turnaround success. Additionally, we proceeded with a cluster analysis which is a multivariate method of data classification carried out by separating the data into groups, called clusters. Finally, an ANOVA test was implemented to find the most significant variables of these clusters.

All the statistical tests were performed at the statistical significance level of 5%. Data were analyzed using SPSS software, version 22 (Statistical Package for Social Sciences Inc., 2003, Chicago, USA).

## Results

### Descriptive statistics

Having in mind the methodology we proposed before, in this epigraph we show the most relevant results.

Most of the participants were males (N = 101, 67.3%) while N = 49 (32.7%) were females. In terms of age, the majority ranged from 40 to 49 years of age (N = 73, 48.3%), followed by the age group 50 to 59 (N = 39, 25.8%). Regarding the employees’ educational level, sector and size of the company, we can see it in Table 1.

**Table 1:** Educational level, sector, and size of the company

**Highest education qualification**	**N**	**%**
**Primary level**	2	1.2
**High School**	1	0.7
**University**	55	36.2
**MSc/MBA**	86	56.6
**PhD**	8	5.3
**Sector**	**N**	**%**
**Industrial Sector**	75	49.3
**Trading Sector**	77	50.7
**Size of the company**	**N**	**%**
**Small**	51	35.3
**Medium**	34	23.6
**Large**	59	41.1

Respect the impact of crisis on companies, we collect the details in Table 2.

**Table 2:** Impact of crisis on companies

**Please indicate to which extent economic crisis affected on your company generally during 2010 to 2016**	**N**	**%**
**Extremely negative**	45	29.6
**Mostly negative**	35	23.1
**Somewhat negative**	33	21.7
**Neither negative nor positive**	16	10.5
**Somewhat positive**	9	5.9
**Mostly positive**	5	3.3
**Extremely positive**	9	5.9
**During fiscal years 2010 to 2016 your company faced a critical situation which you have had to take extraordinary measures?**	**N**	**%**
**Yes**	107	70.4
**No**	40	26.3
**I don’t know**	5	3.3

During the 2010 to 2016 economic crisis, the companies that participated showed that a 29.6% were extremely negatively affected (N = 45) and 25.8% mostly negatively affected (N = 35). We observe that 74.3% were negatively affected by the financial situation while only 15.1% were positively affected to some extent. Most of these companies had to take extraordinary measures to deal with the situation (N = 107, 70.4%).

We also asked the managers what was more affected during the financial crisis. More specific, we asked the effects on operations-indicators during the country's economic situation, indicating that the strategic plan of their company was mostly affected negatively by 30.5% (Ν = 46), turnover was mostly affected negatively by 29.8% (Ν = 45), the results of the company were affected extremely negatively by 26.5% (Ν = 40), the ROI of their company was mostly affected negatively by 24.2% (Ν = 36) and the ROA of their company was mostly affected negatively by 29% (Ν = 40). Summarizing, the negative answers (extremely negative, mostly negative, somewhat negative) for each individual factor (strategic plan, turnover, results, ROI, ROA), exceeds 60% in all.

As for the indicators that the management was aware of the company’s situation when the crisis took place, the participants mostly agree that the management took into consideration the turnover reduction for more than one year (33.3%, N = 50), but the majority responded that they neither agree nor disagree that the management took into consideration the Profit & Loss statement losses for more than one year (22.7%, N = 34) and the ROI reduction of more than > 10% (27.9%, N = 41). On the other hand, managers at a 78% took into consideration the reduction of their turnover more than one year instead of Profits & Losses. 48% considered that as a signal that their company is in a critical situation. Regarding ROI reduction > 10%, the 50% of the managers gave positive answer that this indicator was for them a signal of the company's downfall. We understand that to a large extent that our findings confirm previous research on the criteria showing that the company is in decline phase.

According to the literature review and theoretical knowledge we can argue that companies decline due to internal and external factors. For this reason, we asked the Greek managers to tell us if they believe that their company crisis is due to internal or external factors. 57.3% of the sample believe that the crisis resulted both due to internal and external factors with 23.8% (Ν = 35), believing that such factors played an extremely important role. Regarding the external factors, participants declared that the government’s strategy and restrictions (69.7%, N = 106) and changes made to the needs/demands of the market (65.1%, N = 99) affected their company situation whereas as for the internal factors participants claimed that the very high operating costs (labor costs, energy, etc.) (57.2%, N = 87) and the lack of liquidity, cash flow problems (48%, N = 73) affected their company situation.

We tried to see to what extent they believe that every single factor impacts their company situation. This question is to show us how strongly they believe the answer that they gave before.

Regarding external factors, we see that 50% believe that the reduction of GDP plays an important role, 65% that the changes that occur in the needs of the market also affect. Near 70% believe that the government's strategy and the constraints imposed by the government are a key external factor, while 42% and 54% respectively said that the credit policy of suppliers and the insolvency of customers had a negative effect. On the contrary, the reduction of wages and the increase of unemployment did not exceed 40% of the positive answers.

The internal factors that prevailed with the highest percentages in the answers of the respondents were the high operating costs as labor costs, energy, etc. (57%), the lack of liquidity and cash flow problems (48%), the strong internal bureaucracy that causes delays in the implementation of new projects (34.2%), the blurred internal communication between executives (29%), and the wrong strategy (19%).

We also study about the tactics managers tried to implement to reverse the situation or to prevent to become in crisis. In this sense, 40.1% (N = 61) of the participants affirm that their company did not increase the prices of their products/services, whereas only 6.6% (N = 10) claimed that their company increased the prices with great ease (16.6%, Ν = 25). However, the participants also claimed that their company neither reduced the prices of their products/services (19.1%, Ν = 29). Most of the companies had to give discounts to their customers to be paid in advance/cash (24.3%, Ν = 37) which was neither easy nor hard (22%, Ν = 33). Many companies neither removed non profitable products/services (24%, Ν = 16), nor non profitable distribution channels (32%, Ν = 48). Moreover, the companies were not forced to proceed with personnel wages/salaries cuts (41.3%, Ν = 62) nor were they forced to reduce headcounts (41.7%, Ν = 63). 16% (N = 24) of the companies negotiated and managed to reduce raw materials, services cost of their suppliers including electricity, etc., 10.7% (N = 16) of the companies negotiated and managed to reduce/take discounts for their real estate rents. 11.6% (N = 17) of the companies negotiated with banks or other debtors to reduce somehow the financial cost of the company and 81.6% (N = 124) of the companies succeed in reducing operating costs. 25.8% (N = 39) of the companies did not make any changes at their top management. 23.8% (N = 35) of the companies did not implement any reductions with their promotion and marketing activities and 59.3% (N = 89) did not implement a divest plan, for example a sale of fixed assets, a sale of subsidiaries, etc. Finally, the companies managed to succeed in increasing their cash flow liquidity (63.8%, N = 97) and increase their turnover (59.9%, N = 91).

Additionally, our findings showed that 60% managed to increase their turnover in contrast to the 40% that did not achieve a turnover increase. About 64% of the companies improved their liquidity situation and 92% did not remain at a loss while only 30% improved their RΟΙ. After implementing turnaround tactics, most of the companies 81,6% managed to reduce their operating cost.

Most of the companies had implemented precautionary measures to avoid a future crisis due to country’s general economic situation (80.4%, N = 115). Furthermore, 83.6% (N = 127) of the companies had taken extra measures to reverse the situation affected by the crisis or to avoid a crisis.

Following the theory and literature review and relying more on the turnaround model of Bibeault (1982), it is concluded the rational steps application sequence of some turnaround tactics:

1^st^ situation analysis, 2^nd^ reshuffling, changing top management, 3^rd^ reform middle managers –improvement of communication strategy – actions to change corporate culture, 4^th^ taking control – performing critical cash management, 5^th^ start operating – cost reduction measures, 6^th^ selling assets and surplus inventories, 7^th^ restructuring liabilities, 8^th^ Revisiting business strategy and considering divestments, 9^th^ Development – market and sales focus improving sales and marketing.

To understand what the managers of Greek companies know about the design of a turnaround process, in our questionnaire we asked them to indicate from 1 to 9 successively how they should be applied. In this sense, we received the following answers: 1^st^ situation analysis 47.6, 2^nd^ reshuffling changing top management only 4,1%, 3^rd^ reform middle managers –improvement of communication strategy – actions to change corporate culture 7.6%, 4^th^ taking control – performing critical cash management 14.9, 5^th^ start operating – cost reduction measures 13.5%, 6^th^ selling assets and surplus inventories 12.7%, 7^th^ restructuring liabilities 8.1%, 8^th^ Revisiting business strategy and considering divestments 14.9%, 9^th^ Development – market and sales focus improving sales and marketing 18,9%. We observe that regarding the first step most of the responders agree that must be the situation analysis. As for the second step which is 2^nd^ only the 4% agree on that instead and with approximately the same percentages the application of this measure is ranked after the third position. Obviously, it is quite difficult for the managers themselves to admit that there must be a change in management. The same is happening for the 4^th^ tactic regarding middle management. Concerning the 5^th^ step the 13.5% place it in the right order but the majority consider that it must be 2^nd^ in the row. This is not so wrong following other models. Selling assets placed from most of our responders at the 7^th^ and 8^th^ place and it is rational because there is an emotional bonding with company assets, and everybody is trying to avoid it. In any case, of course, selling assets is not a bad tactic and sometimes it is necessary to be implemented. Speaking for restructuring liabilities the majority place 3 to 6^th^ in the row. For the business strategy the majority consider that it must be at 3^rd^ place which is not right. Finally, regarding the development of the company focusing on marketing and sale, the majority answered that it must be at the 1^st^ and 2^nd^ position against the 18.9% which place it in the right row.

### Statistical analysis

A chi-square test was used to examine the association between the external factors that affected the company situation and the degree to which the operations-indicators were influenced by these factors (p < 0.05).

We find that there was a close relationship between GDP reduction and the company's strategic plan. 39.9% who consider that GDP is an important factor for the situation of their company state that its strategic plan was negatively affected as opposed to 29.2% who believe that the strategic plan was affected positively.

The difference of 10.7% is statistically significant and confirms that the decrease in GDP had a negative impact on the company's strategic plan. The difference of 0.1% between those who said that their strategic plan was negatively affected, in relation to those who said that it was positively affected and believe that changes to market needs is an external factor responsible for the crisis of companies is not statistically significant. The government strategy factor where 39.4% of those who believe that it had a negative effect on the strategic plan have a difference of 15.2% compared to those who said that it had a positive effect. The same happens with the suppliers’ reduction of credit policy with a difference of 8% between negative and positive and with the insolvency of your customers with a significant statistical difference of 11.9%. On the contrary, those who stated that reduction of wages and unemployment increase are external factors of crisis stated that their strategic plan was positively affected by significant statistical differences.

A Pearson chi-square test was performed to detect which external factors influence a business based on the degree of agreement for the following factors. Regarding the answers given by the participants of the survey as to the extent the GDP reduction effected the crisis, 77.6% of the participants extremely agreed statistically significant (p-value < 0.001). The participants who claimed that changes in the market were an external factor influencing the company’s situation, 54.8% of these participants extremely agreed statistically significance (p-value < 0.001) of its impact. Government strategy and restrictions being another external factor was supported by 84% of the participants who extremely agreed statistically significant (p-value < 0.001). The participants who supported that the reduction or stop of their suppliers’ credit policy were an external factor 60.9% extremely agreed statistically significant (p-value < 0.001). As for customer insolvency 69.5% of the participants extremely agreed statistically significant (p-value < 0.001) that it had an impact on the company’s situation. Regarding, reduction of wages and pensions 66.7% of the participants, extremely agreed statistically significant (p-value < 0.001) that it was an important external factor. Finally, 69.6% of the participants extremely agreed statistically significant (p-value < 0.001) that the increase of unemployment was a major external factor.

Regarding the answers given by the participants of the survey as to the extent that the company didn’t follow market need/trends 44,4% extremely agreed statistically significant (p-value < 0.001), the company wrong strategy 53,6% extremely agreed statistically significant (p-value < 0.001) and that no investments implemented in new products/ services 46,2% extremely agreed statistically significant (p-value < 0.001); all were internal factors influencing the company’s situation. Moreover, the fact that there was no opening in new markets/exports 65.4% extremely agreed statistically significant (p-value < 0.001), that there was a strong internal bureaucracy that causes delays in implementation of new projects 61.5% extremely agreed statistically significant (p-value < 0.001), an inefficient distributions network 31.3% extremely agreed statistically significant (p-value < 0.001). The fact that there was bad product quality 75% extremely agreed statistically significant (p-value < 0.001) were seen as internal factors that played a vital role in the company’s situation. Other internal factors that were given as influencing the company’s situation were, inappropriate services where 54.5% of the participants extremely agreed statistically significant (p-value < 0.001), blurred internal communication between executives 33.3% extremely agreed statistically significant (p-value < 0.001) and lack of liquidity, cash flow problems 71.2% extremely agreed statistically significant (p-value < 0.001). Last but not least, lack of transparency regarding procedures and data 23.1% extremely agreed statistically significant (p-value < 0.001), no clear targets for their employees 45.8% extremely agreed statistically significant (p-value < 0.001) and very high operating costs (labor costs, energy, etc.) 45.3% extremely agreed statistically significant (p-value < 0.001) were also internal factors that were crucial in having an impact on the running of the company during the economic crisis.

A factor analysis was conducted to reduce the number of initial variables into fewer factors. The variables used were the questions “Please, indicate the extent to which you believe that the following external factors were responsible for the crisis that your company faced” and “Please, indicate the extent to which you believe that the following internal factors were responsible for the crisis situation that your company faced” with possible answers (1) Entirely Disagree to (7) Extremely agree. More specifically, it was found that the data are suitable for conducting the analysis, as the statistical index Kaiser-Mayer-Olkin (KMO) has a value of 0.736, which is considered satisfactory. In addition, according to the Bartlett sphericity test (X2 = 1138.3, p-value < 0.001) there are significant correlations. Table 3 presents the results of the factor analysis, with five estimated factors. The varimax rotation was also used, due to the fact that the extracted factors are not correlated with each other. The five extracted factors explain 55.1% of the total variability of the initial variables. The questions “Changes to the market needs/demands (technologically, services, quality, etc.)”, “Insolvency of your customers” and “Strong internal bureaucracy caused delays in implementation of new projects” were removed from the analysis due to low factor loadings (0.396, 0.278 and 0.392, respectively). Finally, we create 5 factors. Set of factor 1 variables represent “poor management”, set of factor 2 variables represent “Macroeconomics causes” non-controllable from the management team, set of factor 3 variables represent “Lack of development actions”, set of factor 4 variables represent “internal economic problems” lack of cash and high operating cost and finally factor 5 represent the “role of the government policy”.

**Table 3:** Factor analysis groups

	Factor				
	1	2	3	4	5
No clear targets for your employees	0.857				
Inappropriate /bad quality of services	0.715				
Blurred internal communication between executives	0.669				
Lack of transparency of procedures and data	0.668				
Wrong Strategy	0.562				
Bad products quality	0.516				
Inefficient distribution network	0.423				
Increase of unemployment		0.925			
Reduction of wages and pensions		0.838			
GDP Reduction		0.527			
There was no opening up to new markets and export growth			0.727		
No investments were implemented in new products and services			0.642		
The company did not follow the new market trends/needs			0.512		
Very high operating costs				0.790	
Lack of liquidity, cash flow problems				0.614	
Government strategy and restrictions such as insufficient and highly taxations capital controls etc					0.728
Reduction or stop of your suppliers’ credit					0.374

We used Spearman correlation coefficients for the examination of the following question “Please, indicate the extent to which you believe that country economic situation affected negatively to the following operations-indicators” and the extracted factors of the analysis (Table 3).

Significant negative relationships were observed between factor 3 and the following variables: strategic plan (r = -0.241, p-value < 0.01), turnover (r = -0.248, p-value < 0.01). Results factor 4 was negatively correlated with the strategic plan (r = -0.442, p-value < 0.01), the turnover (r = -0.513, p-value < 0.01), results of the company (r = -0.458, p-value < 0.01), the ROI (r = -0.457, p-value < 0.01) and the ROA of the company (r = -0.411, p-value < 0.01). Similar results were also found for the factor 5 which was negatively associated with the strategic plan (r = -0.299, p-value < 0.01), the turnover (r = -0.232, p-value < 0.01), results of the company (r = -0.306, p-value < 0.01), the ROI (r = -0.299, p-value < 0.01) and the ROA of the company (r = -0.277, p-value < 0.01).

We also did a cluster analysis. The k-means procedure generates the clusters. In the first cluster were classified the companies that had a somewhat negative impact on their financial situation due to the economic crisis, took extraordinary measures to deal with it and, as for the indicators that took into consideration, seemed to be neutral for indicator “Turnover reduction for more than one year”, disagree with the indicators “P&L losses for more than one year” and “ROI reduction of more than > 10%”. In the second cluster were classified the companies that had a mostly negative impact on their financial situation, took extraordinary measures to deal with it and as for the indicators that took into consideration, seemed to be mostly agreed with all the three indicators and mostly with the “Turnover reduction for more than one year”. Tables 4 and 5 show the cluster analysis.

**Table 4:** ANOVA analysis to find the most significant variables into clustering procedure

	Cluster	
	1) somewhat negative impact	2) mostly negative impact
Please indicate to which extent economic crisis affected on your company generally during 2010 to 2016	3.3	2.2
During fiscal years 2010 to 2016 you company faced a critical situation which you have had to take extraordinary measures?	1	1
Please, indicate the extent to which you believe that your management was aware and confirmed a crisis in your company taking into consideration the following indicators:		
*a) Turnover reduction for more than one year*	4.3	6.1
*b) P&L losses for more than one year*	2.9	5.7
*c) ROI reduction of more than* > *10%*	3.5	5.5

**Table 5:** ANOVA to test the significance of variables

_ANOVA_						
	_Cluster_		_Error_		_F_	_p-value_
	_Mean Square_	_df_	_Mean Square_	_df_		
_Please indicate to which extent economic crisis affected on your company generally during 2010 to 2016_	_42.118_	_1_	_2.559_	_145_	_16.461_	_<0.001_
_During fiscal years 2010 to 2016 you company faced a critical situation which you have had to take extraordinary measures?_	_0.932_	_1_	_0.290_	_145_	_3.214_	_0.075_
_Please, indicate the extent to which you believe that your management was aware and confirmed a crisis in your company taking into consideration the following indicators:_						
_*a) Turnover reduction for more than one year*_	_118.448_	_1_	_1.346_	_145_	_88.012_	_<0.001_
_*b) P&L losses for more than one year*_	_292.768_	_1_	_1.365_	_145_	_214.511_	_<0.001_
_*c) ROI reduction of more than*>*10%*_	_156.003_	_1_	_1.232_	_145_	_126.647_	_<0.001_

Following Table 5, the means of the following clustering variables’ “Please indicate to which extent economic crisis affected on your company generally during 2010 to 2016”, “Turnover reduction for more than one year”, “P&L losses for more than one year” and “ROI reduction of more than > 10%” differed significantly between the two clusters (p-value < 0.001), indicating the great influence in the forming of clusters. Whereas the variables “During fiscal years 2010 to 2016 you company faced a critical situation which you have had to take extraordinary measures?” has the least influence, as it was observed that the means did not statistically different between the two clusters (p-value = 0.075).

## Conclusions

The term crisis means the reduction of economic activity in general when it concerns the economies of countries, meaning the negative performance of all macroeconomic indicators such as GDP, unemployment, investments, etc. In companies and organizations, by the term crisis we mean the reduction of its turnover, the reduction of its production, the low efficiency of indicators such as ROI and ROA which leads to redundancies, lack of liquidity, inability to meet its obligations, etc. The crisis often varies based on the causes of its occurrence and the general environment of economic activity. Such crises often threaten even the survival of companies and lead to the termination of their operations. Business crises are due to external and internal factors. With external factors we mean factors that may affect the operation of the business, but managers can not intervene for example GDP reduction, government intervention and government restrictions, high taxes, etc. Internal factors are those that come from the internal operation such as poor management, poor strategic decisions, and high operating costs.

Based on the literature, we investigated in detail external and internal factors but also others that are not mentioned frequently in the literature such as government restrictions, capital controls, etc. The external factors that can negatively affect the operation of the company and lead it to decline are easier to identify than the internal factors where the company's management not always admits being responsible for the situation where the company was found.

As a recovery strategy we define a set of actions and tactics for the company to return to a healthy state, to avoid risk, to return to profitability and to ensure the necessary levels of liquidity that will ensure its smooth operation.

From the analysis of the literature, we suggest that a successful turnaround should have the following elements: a) Analysis of the present crisis which entails taking control, performing critical cash management, reducing assets, arranging short-term funding, and initiating cost-cutting measures. b) Proceed in new management analyzing the replacement of the CEO as well as assessing and replacing senior management as needed. c) Stakeholder communication — It is critical to include all stakeholder engagement. d) Strategic review entails revisiting the company's strategy and considering divestment, asset reduction, downsizing, outsourcing, or investment. e) Making structural- cultural changes in the business; reshuffling, changing, or reducing line of middle management. f) Implementing improvements in sales and marketing; further cost reductions and efficiencies. g) Financial restructuring which entails refinancing, asset reduction, and changes to debt and equity. h) Continuing monitoring of the procedure, feedback, and evaluation.

In our research we did an empirical analysis of the crisis and turnaround tactics implemented to understand better turnaround tactics and causes of companies’ decline during the recession phase of the economic cycle. 152 Greek companies participated in our research whose total turnover represents 3% of the Greek PIB.

Regarding the reasons that affected their business, 7.3% answered internal, 35.3% external and 57.4% internal and external, confirming previous papers that have been written that it is very difficult for managers to admit that only internal factors are responsible for the fact that their business is in a critical situation, wanting to relinquish their responsibilities.

Between the external factors, the most negative was the government strategy and the government restrictions with 69.7%. The main internal factors that negatively affected were the high operating costs, internal bureaucracy which delayed the implementation of projects, and certainly the lack of liquidity which literally strangled their normal operation.

Regarding the tactics applied, our findings showed that 40.1% did not proceed with any increase in the prices of their products in contrast to 59.9% where they proceeded with small increases to large increases. On the contrary, we wanted to see if they proceeded to reduce their prices and we found that only 20.1% did not proceed with any price reduction while 79.9% reduced the prices of some products. It is also important to mention that 86.8% applied discounts to their customers from small to very large discounts while only 13.2% did not give any discount.

The Greek managers implemented measures to reduce their operating costs as well as conducted negotiations with various creditors and banks to reduce their capital costs and restructure their liabilities since the 73.3% implemented negotiations from small to a large scale. On changing the top management only 25.8% take no change applying largely the first measure step for a successful strategy turnaround. Our findings showed that after the implementation of the above measures 60% managed to increase their turnover and about 64% of the companies improved their liquidity situation. The tactics applied seem to have been effective since 81.6% of the companies managed to reduce their operating costs.

We wanted to check if the implementation of the tactics that were implemented was much easier in the specific period where the cycle of the economy was in recession. Regarding the increase of the prices, we observe that 75% made a limited application of the measure while 25% applied it to a greater extent.

In summary, we see that the economic crisis really affected Greek companies to a great extent, both in terms of their strategy and in terms of their performance. It was realized that very quickly measures were taken by the managers with quite high efficiency, this resulted in stabilization and reduction of operating costs with a direct link to the successful results. What we could not determine with great certainty is whether in the end there is a relation between the ease of application of the turnaround tactics with the phase of the economic cycle and whether it would be much easier to apply them in the recession phase.

If the strategic plan is implemented by the owner or the top management, it does not correlate with whether there are successful results in the implementation of a turnaround plan. Greek managers got the knowledge of turnaround tactics which must apply in a crisis but not every one of them has the same opinion about the order of the implementation of each one. With great certainty retrenchment phase is a priority for them.

A factor analysis was conducted to reduce the number of initial variables into fewer factors. As a general conclusion of this test, we can affirm that the more extent the internal or external factors were responsible for the crisis of the company, the more negatively affected the strategic plan, the turnover, the financial results, the ROI and ROA of the company.

Cluster analysis was developed. This is a multivariate method of data classification carried out by separating the data into groups. The k-means procedure generates two clusters. In the first cluster were classified the companies that had a somewhat negative impact on their financial situation due to the economic crisis, took extraordinary measures to deal with it and as for the indicators that took into consideration, seemed to be neutral for the indicator “Turnover reduction for more than one year” and disagree with the indicators “P&L losses for more than one year” and “ROI reduction of more than > 10%”. In the second cluster were classified the companies that had a mostly negative impact on their financial situation, took extraordinary measures to deal with it and as for the indicators that took into consideration, seemed to be mostly agreed with all the three indicators and mostly with the “Turnover reduction for more than one year”. “Turnover reduction for more than one year”, “P&L losses for more than one year” and “ROI reduction of more than > 10%” differed significantly between the two clusters (p-value < 0.001), indicating the great influence in the forming of clusters.

The originality of this paper is based on the development of a comprehensive theoretical framework that examines the factors that influence strategic plan, turnover, and results of an organization during a recession phase of the economy. These factors were tested empirically through research contacted for companies operated in Greece which faced unprecedented conditions. Additionally, this research develops an integrative theoretical framework that combines a set of factors that influence Greek companies. The study aims to investigate the effects of economic crisis in Greece on the business operating in Greece, how the Greek managers tried to overcome this crisis which tactics implemented, and which was the ease/difficulty rate of the implementation of these tactics. This research is a detailed map of data related how the Greek companies affected by the crisis during this specific period. The contribution to knowledge is that it is a study to report valuable data from Greek organizations and how Greek executives pursue turnaround tactics and address which are those factors that influence their decisions and how these tactics affected on their results. Additionally, the study examines the role on successful turnaround based to whom formulated the strategic plan of the company and investigate the knowledge of the Greek managers on the tactics implemented during a turnaround plan. Finally, we think that offers appropriate data for management practitioners to understand how to implement a turnaround strategy in conditions of very unpleasant environment and which are the right tactics to succeed desirable results.

## Limitations and future lines of research

Respect the limitations of the study, we must recognize that the questionnaire was filled in by a single respondent of each company. Data were collected from a single source. Multiple respondents per firm will be highly recommended in future research to reduce the effects of systematic response bias.

Another limitation is that the sample consists only of companies in industrial and trading sectors and it is not included many others sectors such as services, tourism industry, banks and so on, a fact that implies that we will be unable to make generalizations at the total market. The results are from companies operating in Greece and are not necessarily generalizable to other countries.

We also must add that the variables-answers are examined on a managerial basis and hence have a degree of subjectivity. However, managers have a thorough insight and knowledge of strategies and turnaround tactics.

In relationship with future research, as we mention before, the questionnaire was filled in by a single respondent of each company. Data was collected from a single source. Multiple respondents per firm will be highly recommended in future research to reduce the effects of systematic response bias.

We would also like to point out that it would be interesting to address questions relative with the present research in pandemic conditions such as Covid 19 where the market is underperforming. It’s a situation that has nothing to do with the financial cycle.

Of course, it would be interesting to measure and investigate what happened in other sectors besides the commercial and industrial that this work deals with.

Finally, our research concerns companies operating in Greece. The results and actions implemented include the culture of Greek managers. It would be an important contribution to investigate what happened, what tactics were applied and whether there was a correlation with the economic crisis in other European countries and worldwide.
